# Understanding the Lingual Frenulum: Histological Structure, Tissue Composition, and Implications for Tongue Tie Surgery

**DOI:** 10.1155/2020/1820978

**Published:** 2020-06-28

**Authors:** Nikki Mills, Donna T. Geddes, Satya Amirapu, S. Ali Mirjalili

**Affiliations:** ^1^Pediatric Otolaryngology Department, Starship Children's Hospital, Auckland, New Zealand; ^2^Department of Anatomy and Medical Imaging, Faculty of Medical and Health Sciences, University of Auckland, Auckland, New Zealand; ^3^School of Medicine and Pharmacology, University of Western Australia, Crawley, Perth, Western Australia, Australia

## Abstract

Lingual frenotomy has become an increasingly common surgical procedure, performed for a broad range of indications from birth through adulthood. This study utilizes histology to define the structure and tissue composition of the lingual frenulum and floor of mouth (FOM) fascia. En bloc specimens of anterior tongue, lingual frenulum, and FOM tissues were harvested from ten embalmed adult cadavers. An additional three fresh tissue cadaveric specimens were frozen with the tongue supported in an elevated position, to enable harvesting and paraffin embedding of the elevated lingual frenulum as a discrete specimen. All 13 specimens were prepared as ten-micron coronal sections using stains to determine the general morphology of the lingual frenulum, its relationship to neighbouring structures (Mason's Trichrome), presence of elastin fibers (Verhoeff-van Gieson), and collagen typing (Picrosirius Red). Our results have shown a submucosal layer of fascia spanning horizontally across the FOM was present in all specimens, with variability in fascial thickness and histologic composition. This FOM fascia suspends the sublingual glands, vessels, and genioglossus from its deep surface. The elevated lingual frenulum is formed by a central fold of this FOM fascia together with the overlying oral mucosa with variability in fascial thickness and composition. With tongue elevation, the fascia mobilizes to a variable extent into the fold forming the frenulum, providing a structural explanation for the individual variability in lingual frenulum morphology seen in clinical practice.

## 1. Introduction

The lingual frenulum is of significant clinical relevance because of its potential to restrict tongue mobility. The frenulum has traditionally been described as a mucosal fold, which can restrict tongue mobility by tethering to the mandible or floor of mouth (FOM) [1]. Over the last decade, there has been increasing popularity in the frenulum being conceptualized as a discrete submucosal connective tissue midline band or “string” [2]. However, the understanding of lingual frenulum structure has been comprehensively revised following two studies using microdissection of fresh tissue cadavers [3, 4] showing that the lingual frenulum is formed by a midsagittal fold in a layer of fascia that spans across the floor of mouth.

Only two published articles on the histology of the human lingual frenulum exist but neither provide a comprehensive understanding of frenulum morphology or structure [5, 6]. In 1966, Fuchs reported on histological analysis of 25 lingual frenulums harvested from cadavers aged 1 to 70 years old [5]. Nonkeratinized squamous epithelium was described covering the lingual frenulum, in direct continuation with the epithelium from both sides of the FOM as well as that of the ventral surface of the tongue. Connective tissue fibers beneath the epithelium in the region of the lingual frenulum were described as crossing diagonally to the longitudinal axis of the frenulum, forming a scaffold-like framework. Despite these findings, Fuchs described the frenulum structure as a “band.” This discrepancy in interpretation was possibly due to their study assessing excised frenulums, excluding the possibility of understanding the in-situ histotopography of the frenulum.

Almost 50 years later, in 2014, Martinelli et al. analyzed tissue specimens excised during frenulum surgery in 7 children under 4 years of age [6]. Skeletal muscle fibers were identified in some specimens. Collagen fibers were reported as being predominant Type I in all specimens, with Type III collagen fibers usually located near the epithelium and around blood vessels. Variability in the abundance and location of elastin fibers was also noted. The location, size, and orientation of the biopsied tissue were not stated, and therefore, no conclusions were able to be drawn regarding frenulum structure or morphology.

Histological analysis of the sublingual fascial layer in Rorqual (Balaenopterid) whales revealed collagen and abundant elastin fibers loosely organized into randomly oriented fibers forming three distinct layers [7]. It was hypothesized that the whales' sublingual fascia has a role in facilitating the gliding movement of the tongue relative to adjacent tissues during swallowing. These findings provoke curiosity regarding the histotopographical composition of the human lingual frenulum and how its structure and composition may correlate with a role in balancing tongue mobility and stability during functional tasks.

This study aims to describe the histological composition and architecture of the layers forming the lingual frenulum. Using adult human cadavers, we assess the variability in these features between individuals, including an understanding of the frenulum's relationship to genioglossus and FOM structures and the relative mobility and gliding of these layers/structures when tongue elevation is utilized to form the frenulum. We aim to verify if the FOM fascia is consistently present as a histologically identifiable layer, to clarify the connective tissue composition of the FOM fascia, and to establish any variability between individuals.

## 2. Materials and Methods

The 13 adult cadavers (10 embalmed and 3 nonembalmed) used in this research were donated to the Anatomy Department. Ethical consent was obtained under the Human Tissues Act 2008. Basic demographic data is outlined in Table 1.

The tissues were harvested using 2 different techniques.

### 2.1. Histology of the Lingual Frenulum and FOM in 10 Embalmed Cadavers

**Figure 1:**
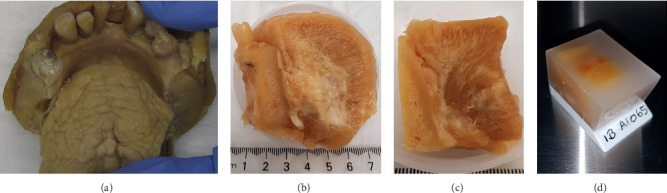
Preparation steps for harvesting and preparing embalmed cadaver specimens. (a) En bloc harvesting of anterior tongue and floor of mouth with central mandible. (b) Specimen trimmed and prepared in formic acid. (c) Specimen trimmed further prior to dehydration in alcohol. (d) Prepared specimen embedded in paraffin.

The central mandible, FOM, and anterior tongue were removed en bloc using the following steps:

- Lateral bony incisions were made on either side of the mandible body
- Soft tissue incisions were made to remove “en bloc” the anterior tongue and floor of mouth soft tissues together with the central mandible (Figure 1(a))
- Preparation in 10% formic acid (replaced every 14 days for 9 months) until sufficient decalcification of bone and teeth had occurred (Figure 1(b))
- Further soft tissue trimming in necessary, making sure the ventral tongue surface and anterior FOM were maintained intact (Figure 1(c))
- Dehydration with ethanol, clearing in chloroform, and then paraffin impregnation to form blocks (Figure 1(d))

### 2.2. Histology of Excised Lingual Frenulum in 3 Fresh Tissue (Nonembalmed) Cadavers

**Figure 2:**
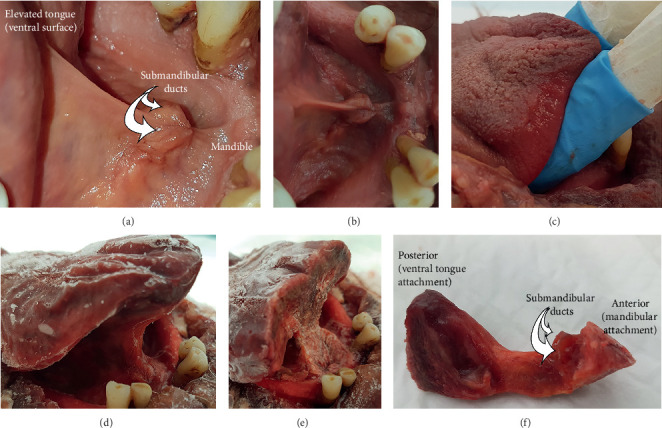
Preparation steps for excising frenulum specimens (fresh tissue cadavers). (a, b) Fresh specimen: tongue elevated to place frenulum under tension. (c) Fresh specimen: tongue supported, keeping frenulum under tension and elevated. (d) Frozen specimen: supports removed , frenulum remains in elevated position. (e) Main tissue block with frenulum excised. (f) Excised frenulum specimen.

It is recognized that the lingual frenulum changes in morphology, becoming more visibly prominent when the tongue is elevated. To assess the morphology of the frenulum in this elevated position, this component of the research used fresh, pliable cadaveric tissue, allowing the tongue to be passively elevated and the lingual frenulum harvested whilst under tension using the following steps:

- The tongue was elevated, and frenulum photographed (Figures 2(a) and 2(b))
- Packing was used to support the tongue in this elevated position (Figure 2(c))
- The tissue block was then frozen
- Once frozen, the packing was removed (Figure 2(d)), and the frenulum was harvested en bloc, including overlying mucosa and the underlying anterior genioglossus fibers (Figure 2(e): main block after excision of frenulum, Figure 2(f): excised frenulum)
- Whilst still frozen, the excised frenulum specimens were orientated and pinned to small polystyrene blocks to secure tissue relationships once thawed
- The specimens were then fixed in 10% neutral-buffered formalin prior to dehydration with ethanol and paraffin embedding

### 2.3. Slide Preparation and Staining

Three consecutive coronal sections (thickness 10 microns) were harvested at intervals of 200 microns, starting anteriorly from the inner surface of the central mandible and continuing posteriorly to beyond where the blade of the tongue merged with the FOM. The sections were placed on adhesive slides. Summary of slide staining is as follows (for each cadaver):

(i) Mason's Trichrome (MTC)
  - To differentiate muscle and collagen
  - One slide from every 200-micron interval (average of 20 slides per cadaver, range 8–38 slides)
(ii) Verhoeff's Van Gieson (VVG)
  - To identify the presence and abundance of elastin fibers
  - Minimum 2 slides per cadaver
(iii) Picrosirius Red (PSR)
  - For collagen typing
  - Minimum 3 slides per cadaver

All slides were viewed using a Leica Microscope at magnification power settings: 4x/0.10, 10x/0.22, and 40x/0.65 with images captured electronically using Leica Microsystems AirLab software. Further high-definition images were captured from selected slides using Meta systems V-slide Zeiss Axio Imager Z2 at 20x magnification. The PSR stained slides were also viewed with a Leica DMR microscope using a Nikon digital sight camera with bright field illumination and polarized light at 5x and 10x magnification for collagen typing.

The coronal section slides were analyzed from the anterior most aspect of the FOM, where gingival mucosa separated from its attachment to the inner surface of the mandible in the midline, to posteriorly beyond where the blade of the tongue merged with the FOM to form the body of the tongue. A descriptive analysis was made of specific histological features for each specimen. Collation of these results was used to summarise common findings and histological variation that existed between individuals.

## 3. Results

### 3.1. Histology of the Embalmed Cadaver Specimens

Following analysis of the slides using the three different staining techniques, the histological features of the lingual frenulum and floor of mouth (FOM) are presented, describing the spectrum of variability observed.

**Figure 3:**
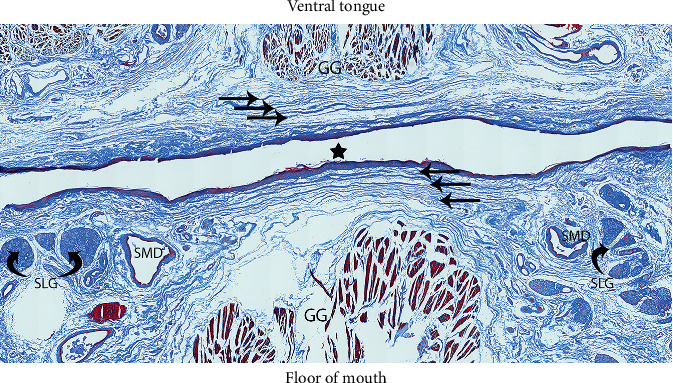
Floor of mouth fascia (MTC stain). Star: midline. Straight black arrows: FOM fascial layers (extending onto ventral tongue surface). GG: genioglossus (muscle fibers). SMD: submandibular ducts. SLG: sublingual glands (curved black arrows).

All specimens had the anterior tongue resting on the floor of mouth, with the FOM epithelial layer and the underlying connective tissues orientated horizontally (Figure 3). As expected, in these embalmed specimens, the fold forming the lingual frenulum was not elevated.

In all specimens, immediately beneath the oral mucosa, there were distinct stratified layers of submucosal connective tissue that spanned horizontally across the FOM (Figure 3). The appearance and composition of these connective tissues are consistent with fascia and therefore are referred to hereon as FOM fascia. The FOM fascia varied from being delicate, thin layers in some individuals to more clearly defined, thicker layers in others. There was no apparent correlation between fascial thickness and either age or gender. There were no discrete connective tissue fibers with a midsagittal orientation (no midline submucosal “cord” or “band”) in any specimen.

**Figure 4:**
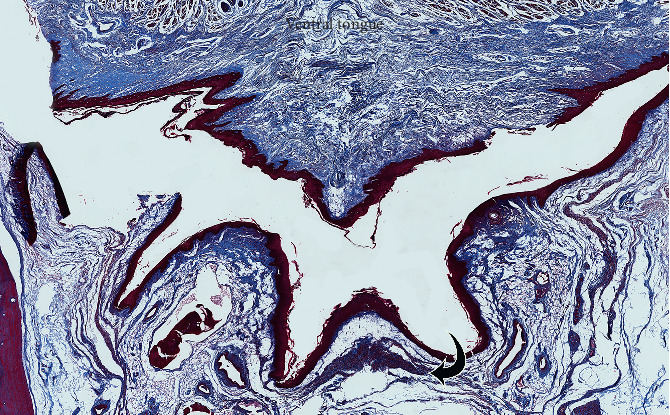
Thickening of central FOM fascia. Thickening of central FOM fascia identified with arrow.

In 8/10 (80%) specimens, there was uniform thickness of the fascia across the whole FOM (i.e., no significant thickening of the fascial layer/s in the midline region of the frenulum) as shown in Figure 3. Notable central thickening of the FOM fascial layer in the region of the lingual frenulum was present in 2/10 (20%) specimens (Figure 4).

**Figure 5:**
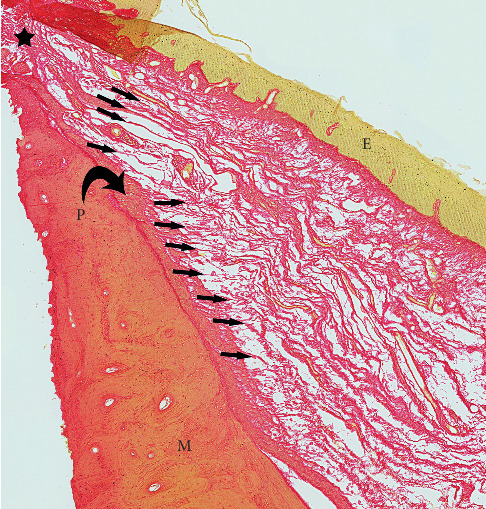
Higher magnification of FOM fascia merging peripherally with periosteum. P: periosteum (curved black arrow). M: mandible. E: epithelium (FOM mucosal layer). Small arrows: FOM fascia merging with periosteum. Black star: lateral floor of mouth where FOM mucosa merges with gingival mucosa.

Peripherally, the FOM fascia merges with mandibular periosteum, “flaring” to attach over a vertically broad area (Figure 5).

**Figure 6:**
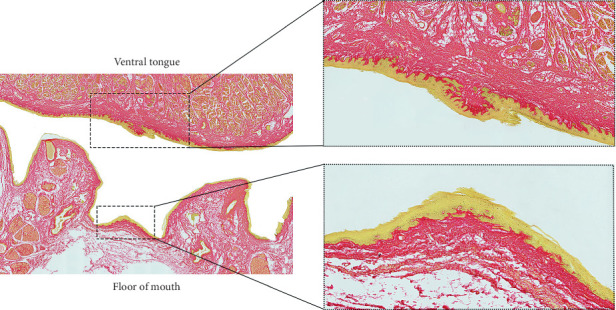
Ventral tongue and floor of mouth–enlargements of midline mucosal surface (PSR stain). Enlargements to illustrate dense connective tissue on ventral tongue and horizontal fascial layers spanning the midline of the floor of mouth between the submandibular duct openings.

Centrally, the FOM fascia merges and is continuous with the superficial dense submucosal connective tissue on the ventral surface of the tongue. The ventral tongue superficial connective tissue was generally thicker and more irregular when compared with the FOM fascia (Figure 6), having dense connections to both the epithelial layer and to the intrinsic muscles that extend to the tongue's surface. There is no direct extension of FOM fibers into the median septum of the tongue.

**Figure 7:**
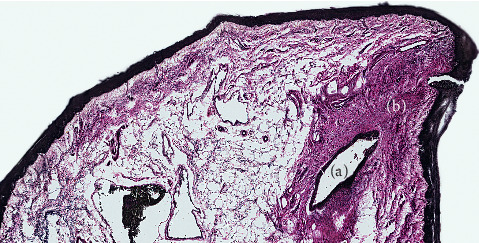
Submandibular duct opening with dense fascia connecting to mucosa. (a) Submandibular duct opening. (b) Dense condensation of fascia suspending duct from mucosal surface.

Genioglossus is suspended from the FOM fascia by a thin vertical layer of connective tissue that is continuous with the epimysial layer surrounding the muscle. On either side of the midline, the FOM fascia separates into layers that envelop or suspend the sublingual glands, the submandibular ducts, and the FOM vessels, with the layers merging together again lateral to these structures. The FOM fascia is dense and thickened around the openings of the submandibular ducts, adhering the ducts to the mucosal surface either side of the midline (Figure 7).

**Figure 8:**
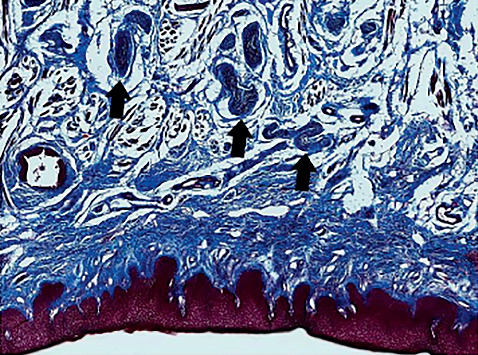
Ventral tongue showing superficial location of lingual nerve branches under higher magnification (MTC stain).

Lingual nerve branches were located superficially on the ventral surface of the tongue, directly under the fascial layer (Figure 8).

**Figure 9:**
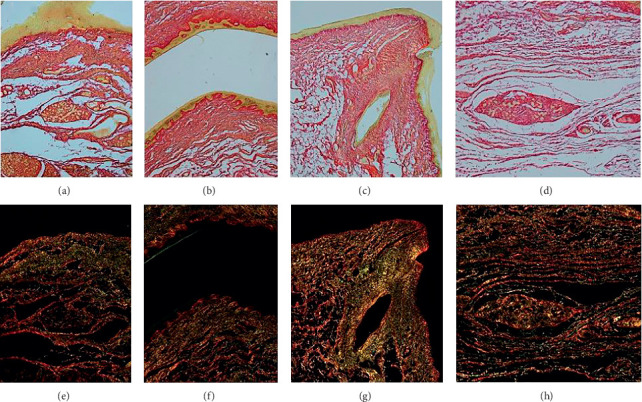
Collagen typing using polarized light microscopy (PSR). (a–d) Bright light microscopy: types I and III collagen stain red. (e–h) Corresponding images using polarized light microscopy: type III collagen highlights green.

The FOM connective tissue was identified to have a high proportion of Type III collagen, shown as green on polarized light imaging of PSR stained slides (Figure 9). Type III collagen was of the highest density in the midline FOM in the location of the lingual frenulum and was less prevalent in the lateral aspects of the FOM fascia.

**Figure 10:**
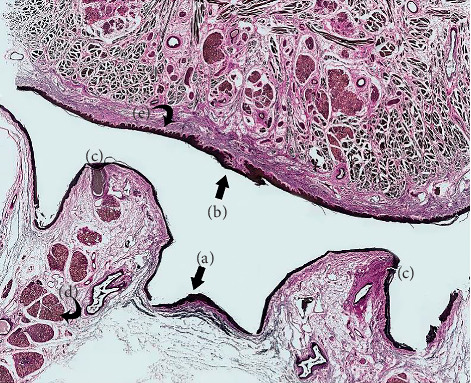
Elastin fibers: whole slide section (VVG). (a) Central floor of mouth (region of frenulum). (b) Ventral tongue (central ridge at superior-most aspect of lingual frenulum fold). (c) Submandibular duct openings (with dense connective tissue surrounding and tethering to mucosa). (d) Sublingual glands (embedded in and suspended from inferior layers of floor of mouth fascia). (e) Ventral tongue: rete pegs at interface between mucosa and connective tissue.

The abundance of elastin fibers and thickness of the elastin layers varied significantly between specimens. Discrete elastin layers were clearly delineated within the FOM fascia, tending to be thin layers in the lateral FOM, forming a thicker layer or layers in the midline region of the frenulum (Figure 10).

The study intentionally did not include quantification of proportions of collagen types or elastin fibers. As there was significant variability in proportions of these fibers across the floor of mouth within every individual, it was felt that any attempt to quantify the amount of any specific fiber type would be confounded by a potential sampling error.

### 3.2. Histology of Excised En Bloc Lingual Frenulum (3 Fresh Frozen Cadavers)

#### 3.2.1. Specimen 1

**Figure 11:**
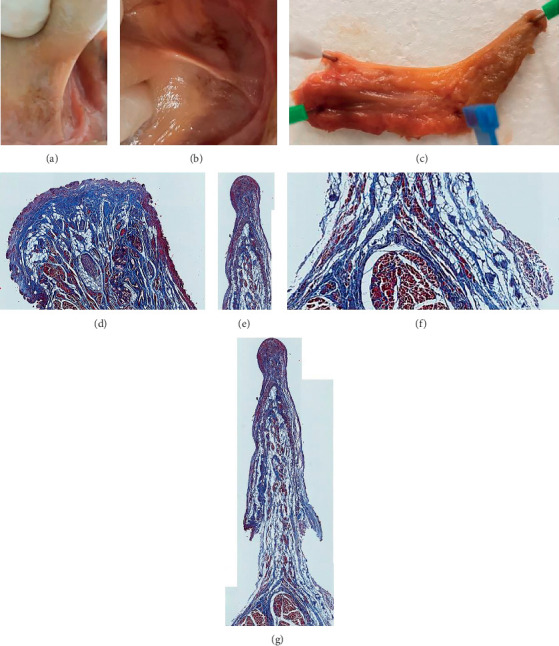
Coronal section of excised lingual frenulum under tension (specimen 1). (a) Frenulum under tension (tongue elevated). (b) Frenulum under tension (tongue retracted). (c) Excised frenulum (white pin: ventral tongue attachment of frenulum). (d) Frenulum at mandibular insertion (anterior). (e): Mid frenulum: fascial layer drawn up into fold with overlying oral mucosa. (f) Suspension of genioglossus. (g) Mid frenulum.

This frenulum was a moderately thick and opaque fold (Figures 11(a) and 11(b)). On histology, the frenulum is shown to be formed by FOM fascia raised into a fold, elevated almost to the full height of the frenulum with the overlying oral mucosa thickened on its superior-most aspect (Figures 11(d), 11(e), and 11(g)). Genioglossus is suspended from an extension of the muscle's external myofascial layer that inserted into the deep layers of the FOM fascia (Figures 11(f) and 11(g)). The morphology of the frenulum changed along the length of the frenulum, with Figure 11(d) showing the complex structure at the anterior-most aspect of the frenulum where it broadens at its attachment to the mandible. Figures 11(e)–11(g) show a coronal section of the mid frenulum, with a more well-defined fold formed by the FOM fascia and overlying mucosa.

#### 3.2.2. Specimen 2

**Figure 12:**
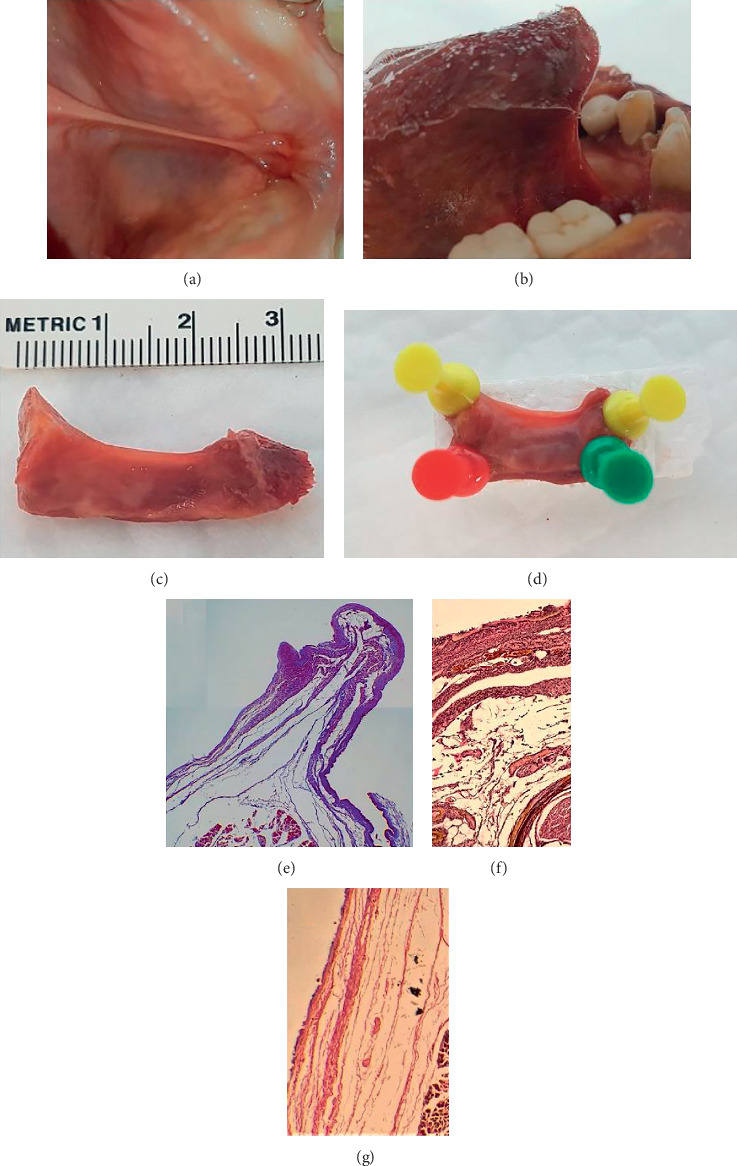
Coronal section of excised lingual frenulum under tension (specimen 2). (a) Frenulum under tension (tongue retracted). (b) Frenulum under tension (tongue frozen in elevated position). (c) Excised frenulum (frozen). (d) Excised frenulum (yellow pins: superior, green: anterior, red: posterior). (e) Mid frenulum: fascial layer drawn up into fold, genioglossus suspended. (f) Mid frenulum: higher magnification to show fascial layer beneath oral mucosa. (g) Submucosal layers of fascia on lateral aspect of frenulum.

When placed under tension with tongue elevation, this frenulum formed a reasonably well defined fold (Figures 12(a)–12(d)). On histology, the frenulum was shown to be formed by thin layers of fascia with the overlying mucosa (Figures 12(e)–12(g)). Genioglossus is shown suspended from the FOM fascial layer (Figure 12(e)), creating a broadening of the inferior aspect of the frenulum.

#### 3.2.3. Specimen 3

**Figure 13:**
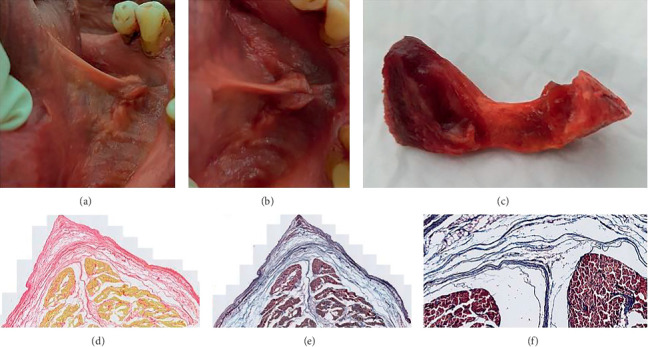
Coronal section of excised lingual frenulum under tension (specimen 3). (a) Frenulum under tension (tongue elevated). (b) Frenulum under tension (tongue retracted). (c) Excised frenulum. (d) Mid frenulum: PSR, genioglossus fibers yellow. (e) Mid frenulum: MTC, genioglossus fibers brown. (f) Mid frenulum: higher magnification of image E.

This frenulum formed an opaque less well-defined fold (Figures 13(a)–13(c)), with coronal sections being broad and almost triangular in coronal section (Figures 13(d) and 13(e)). Genioglossus is drawn up into the fold of the frenulum, suspended close to the deep surface of the fascial layer, with the overlying mucosa elevating into a fold slightly above the layer of the fascia (Figures 13(d)–13(f)).

## 4. Discussion

In all specimens, a submucosal layer of fascia spanning horizontally across the floor of mouth between the inner surface of the mandible and the ventral surface of the tongue was identified. This floor of mouth (FOM) fascia, together with the closely opposed overlying mucosa, forms the “roof” of the sublingual space. Our findings were consistent with the description by Fuchs, with the connective tissue fibers forming a scaffold-like framework [5], but we conclude that the histotopographic structure of the frenulum is definitely not a cord or band, with no discrete midsagittal connective tissue structure identified in any specimen. We observed significant variability in thickness and composition of the FOM fascia between both individuals and across the FOM within the same individual. We did not find any correlation with FOM fascial thickness of composition with age or gender but given our small sample size we cannot exclude that a difference may exist.

With a high concentration of Type I collagen fibers identified in the lingual frenulum biopsies in Martinelli's study, they concluded that the frenulum was resistant to traction, influencing their clinical recommendations regarding surgical management [6]. This is a reasonable assumption, as humans deep and aponeurotic fascia, predominantly comprising of Type I collagen fibers, has been shown to have high tensile strength and low distensibility [8, 9]. However, in contrast to Martinelli's study and using a similar technique for identification, we found a high prevalence of Type III collagen and elastin fibers in the FOM fascia of our specimens. Type III collagen and elastin fibers are usually found in tissues that require greater distensibility and/or mobility, such as those surrounding blood vessels [10]. Interestingly, the sublingual fascia in rorqual whales was shown to have high levels of elastin fibers and had up to 200% distensibility shown on biomechanical testing [7]. In the whales, this distensibility together with features optimizing gliding between fascial layers was thought to facilitate the extraordinary tongue mobility observed in these mammals. We propose that, to a less exaggerated extent, the human FOM fascia has specialized qualities that emulate the functional properties of that described in the sublingual fascia of the rorqual whales. The FOM tissue composition suggests that, in at least some individuals, the FOM fascia may have a greater degree of distensibility than fascia found in other areas of the body, challenging the dogma that all lingual frenulums are nondistensible. However, our study suggests there is significant individual variability in the proportions and distribution of fibers (Type III collagen and elastin) that would influence the fascia's properties of distensibility. We raise the possibility that this variability in tissue composition may impact an individual's range of tongue mobility and biomechanics and may be one reason why individuals with similar frenulum morphology may have variability in whether they experience functional limitation of tongue mobility. However, we acknowledge that in clinical practice it would be difficult to quantitatively measure the distensibility of an individual's FOM fascia.

With our unique preparation of fresh tissue specimens for assessing frenulum histology, we have confirmed that tongue elevation alters the contour of the FOM fascial layer together with the overlying oral mucosa to form the elevated midsagittal fold recognized clinically as the lingual frenulum. There was variability between the specimens in the relative gliding between the mucosal and fascial layers, impacting the morphology of each of the frenulums examined. The mucosa appeared able to elevate into a fold of variable distance above the superior edge of the fascial fold. Genioglossus was also noted to be suspended at a variable distance from the FOM fascial layer, altering the position of genioglossus within the fold of the elevated frenulum and therefore the thickness and contour of the frenulum. These histological findings support the concept proposed in a previous paper [4] for a structural explanation for variability in frenulum morphology.

There is increasing interest in the potential role of fascia when dysfunction of movement is present. However, no anatomy texts, and even those specific to the anatomy of human fascia have not defined, described, or illustrated a layer of fascia in the FOM [9]. Based on its location, histology, and apparent function, the FOM fascia appears to have unique properties and is not easily categorized according to conventional fascia descriptions [11]. As the FOM fascia contains high proportions of Type III collagen and elastin, histologically it has some characteristics consistent with separating or visceral fascia. The FOM fascia appears to also suspend the tongue and floor of mouth structures within the mandible, so we propose the fascia may have a primary role in stabilizing the tongue whilst allowing for optimal range of mobility. This challenges the traditional concept that the floor of mouth is suspended and supported by mylohyoid.

Consistent with the recently described adult and neonatal lingual frenulum anatomy [3, 4], we have confirmed that lingual nerve branches are located superficially on the ventral tongue surface, immediately beneath the fascial layer. This emphasizes the vulnerability of these nerve branches during frenotomy surgery, particularly when using any surgical tool that creates thermal energy that can be transmitted into the underlying tissues. Direct neural connections have been shown between lingual nerve (sensory) branches and hypoglossal nerve (motor) branches [12, 13], creating a direct link for sensation to stimulate intrinsic muscles to alter dorsal tongue contour. Dorsal tongue contouring around the nipple has been shown to be critical for creating the intraoral vacuum required for milk extraction during breastfeeding [14]. Therefore, if temporary or permanent impairment of sensation to the anterior tongue occurred in a neonate at the time of frenotomy, it would be likely to significantly impair their ability to breastfeed.

The major strength of this study is the use of both embalmed and fresh tissue cadavers to analyze the structure of the lingual frenulum. The fresh tissue samples allowed us to assess the histotopography of the frenulum in an elevated position under tension. This novel approach has built on a new structural explanation of clinical variability in lingual frenulum morphology and the dynamic changes in frenulum shape that occurs with altered tongue position.

A weakness of this study is that immunohistochemistry was not used for collagen typing, predominantly because of the expense of doing this form of testing. We refer to research that suggests MTC and PSR stains are sufficient for qualitative testing of collagen content and typing [15] but acknowledge that immunohistochemistry would be required to make any quantitative assessment of collagen fibre typing. As there was significant variability in proportions of collagen types and elastin fibers across the floor of mouth in every individual, it was felt that any attempt to quantify could also be confounded by a potential sampling error. As our study was of small numbers and the tissue was attained from cadavers over 50 years of age, we were insufficiently powered to determine the presence of any gender or age-related changes occurring in fascial thickness that have been suggested in other studies [16].

## 5. Conclusion

The lingual frenulum is shown to be a dynamic structure formed by a midline fold of the FOM fascia together with the overlying FOM mucosa. We have confirmed that the lingual frenulum is definitely not a discrete midline structure. There is significant individual variability in histological composition and microanatomical structure of the FOM fascia. A high proportion of Type III collagen and elastin fibers in the central FOM fascia suggests that, at least in some individuals, the FOM has a composition that allows distensibility of the fascial layer. As tongue elevation creates tension in the FOM fascia, raising the fold of the lingual frenulum, the variability in relative gliding and mobilization of the mucosal and fascial layers appear to impact frenulum morphology. Further biomechanical research is needed to define the impact of these and other variables on tongue mobility and function.

## Figures and Tables

**Table 1:** Cadaver details.

Technique	Cadavers	Numbers	Preparation for histology	Age range (average) years	Male: female
A	Embalmed	10	Anterior tongue “resting” on FOM No tension on frenulum	54–96 (85)	5:5
B	Fresh tissue	3	Tongue frozen in elevated position for harvesting of frenulum	64–85 (78)	1:2
	TOTAL	**13**		54–96 (83)	6:7

## Data Availability

The findings from this study are predominantly qualitative and descriptive and are provided in the manuscript. Any requests for further information to support the findings of this study can be requested from the corresponding author.
